# Safety and Traceability in Patient Healthcare through the Integration of RFID Technology for Intravenous Mixtures in the Prescription-Validation-Elaboration-Dispensation-Administration Circuit to Day Hospital Patients

**DOI:** 10.3390/s16081188

**Published:** 2016-07-28

**Authors:** María Martínez Pérez, Guillermo Vázquez González, Carlos Dafonte

**Affiliations:** 1 Department of Information and Communications Technologies, Faculty of Computer Science, Campus Elviña S/N, University of A Coruña, E-15071 A Coruña, Spain; dafonte@udc.es; 2 A Coruña Universitary Hospital, Xubias de Arriba 84, E-15006 A Coruña, Spain; guillermo.vazquez.gonzalez@sergas.es

**Keywords:** RFID, traceability, safety, adverse effects

## Abstract

This work presents the integration of the RFID technology with the aim of ensuring the traceability of patients and minimization of adverse events during the process of prescription-validation-elaboration-dispensation-administration of medication by means of the implementation of various passive and active WIFI RFID systems in the Pharmacy and Day Hospital services of the Complejo Hospitalario Universitario A Coruña. Obtaining patient traceability and using the patient/drug binomial during this process allows us to minimize the occurrence of adverse events. The key points in this work are the unmistakably unique identification and accurate real time location of the controlled items (patients and medication). RFID technology has proved to be invaluable in assisting with the everyday clinical practice of a hospital, and has been successfully implemented in this environment and others. In services such as the day hospital, the implementation of said technology is further justified by the high costs of the service and the high risk to the patient.

## 1. Introduction

The concern of medical professionals regarding patient safety in the assistance process is nothing new: a robust public healthcare service has many positive effects on the population including wealth generation, social cohesion, increased productivity, consumption growth, and increased research. The public healthcare system is a source of social and economic wealth that creates jobs and plays an important role in preventing premature mortality by increasing and improving the quality of life of citizens.

Patient safety [1,2] is one factor that indicates the quality status of health services and is considered a priority in healthcare. Maintaining patient safety is an increasingly complex task, as it involves potential risk and there is no method that can guarantee the absence of errors. It should be noted that, in healthcare tasks, there is a combination between factors inherent to the environment and human actions [3,4,5,6], and this is a possible cause of what are called adverse events (all situations that occur during the patient care process which do not originate in the underlying health condition but can cause significant damage or even death), which are possibly caused by a combination of environmental factors and human actions [3,4,5,6]. 

Traceability of patients and medications may be even more important for those drugs that are considered high risk and cost since, in addition to the perfect dosage, it greatly facilitates the sustainability of the public health system. This paper mainly focuses on three types of intravenous mixtures: tocilizumab, abatacept, Remicade^®^ (infliximab) and Inflectra™ (biosimilar infliximab), dosified in the Pharmacy Service and administrated at the Day Hospital of A Coruña University Hospital (CHUAC), prescribed for rheumatological, neurological, or digestive diseases. The development of treatments is made according to the pharmacotherapeutic profile of the patient.

Currently, a significant portion of the drugs that are administered to patients in the Day Hospital are prepared and dosed by the Pharmacy Service. These processes are protocolized and, prior to implementation, a record of the medication that is prepared for each patient (indication, dose, pattern) is made. Additionally, for each medication prepared, the lot and expiry date of each of the components, production date, and expiry of the final product are registered. All these records are made manually on paper and it is difficult to ensure the global traceability of the medication, especially if it has been some time since its preparation. Nevertheless the time of the prescription receipt, the departure from the Pharmacy Service, or its reception at the Day Hospital are not registered.

Moreover, the Day Hospital is characterized by a high rotation of patients whose prescribed medication has long term established patterns (21 days, monthly, etc.). It is important to note that all the medication that is to be included in this work has special characteristics in the process of prescription, preparation, and/or administration to the patient. Usually, when a patient arrives at the Day Hospital he or she has to meet several requirements before the prescribed medication is administrated, i.e.,

1. Having a scheduled administration for that day.
2. Having medical tests with favorable results.
3. Prior medication that has already been given (necessary for the patient to be ready to receive the prescribed treatment, e.g., paracetamol).

When the patient meets the requirements described above, the practitioner requests that the Pharmacy Service prepares the medication, but in daily clinical practice there are certain situations that hinder the patient care process. The main problems detected are:

- Medication is received at the Day Hospital but the patient is no longer in the hospital.
- Medication is lost during transport from the Pharmacy Service at Day Hospital.
- When the drug reaches the Day Hospital neither the patient nor the nurse are notified.

The communication of incidents was realized by means of telephonic real time calls between both services (Pharmacy and Day Hospital) to know the reasons of the problem and to try to solve it.

Prior to this study, when the Pharmacy Service had sent the medication, the key parameters of the intravenous mixture elaboration and administration (as shown in Table 1) could not be seen visually or electronically on a single tag, in a quick and safe way. Therefore, traceability of patient and medication control is needed. 

Another key point in the care process is when the nursing staff has to administer a medication to the patient. It would be ideal to have labelled information containing a practical guide to the conditions of use of said drugs (see Table 1) and the most important data concerning the treatment of the patient and other relevant information. It is also essential to automatically and correctly validate that the right medication is going to be administered to the patient.

It is important to note that in both cases the Day Hospital and the Pharmacy Service are working on the basis of current quality standards such as the FDA Good Manufacturing Practice [7], the Requirements of Good Manufacture and Quality Control of the magisterial prescriptions and official preparations, and are of course on the same level of standards as hospitals with a similar level of environment and complexity. In addition, the CHUAC Pharmacy Service has been certified by ISO 9001:2000 since 2005.

The team of the project decided that the best strategy to diminish the possible resistance to the change on the part of the sanitary personnel, is to involve them during all the phases of the life cycle of the project (analysis, design, implementation and tests). From the beginning of the project, the supervisors of area of the units (Pharmacy, Informatics, Rheumatology, Day Hospital) were present at the meetings. In addition, some managers of this sanitary personnel are researchers of a project PI10/02442 that has financed part of this work. In the case that the medical personnel have to do more activities, we always remind them that the security of the patient comes first. Furthermore, the sanitary personnel learned each of the new steps progressively, which was helpful for their learning process. They could always consult a person concerning their doubts about the project’s working method.

## 2. Objectives and Benefits

The objective of this project is to improve patient safety in the process of prescription-validation-preparation-dosification-dispensation-administration of drugs at the CHUAC Day Hospital. This aim will be achieved by implanting a series of RFID systems in the Pharmacy Service and Day Hospital. These systems identify without mistake the patients, medicines, and the patient-prescribed medicine binomial. In addition, these systems ensure the traceability of the patients. The Real Time Location System calculates the location of the patients in real time. This work also facilitates the traceability of the intravenous mixtures, since they are prepared in the Pharmacy Service until finally they are administered to the patients in the Day Hospital. 

The main benefits of this work are: (1) minimizing the occurrence of adverse events, as developed RFID systems facilitate the accurate identification of the patient and the correctly prescribed medication; and (2) ensuring that nursing staff will administer the correct medication in the proper administrative conditions. Various health professionals will be able to check the location of the patients and medication in real time, detecting potential bottlenecks and thereby significantly improving the efficiency of the hospital.

It is important to note that the CHUAC consumption of medication selected for this work and considered high risk, represents an annual cost estimated at approximately four to five million Euros (2014 data obtained through Cost Management and Consumption of Drugs, applications available in the Pharmacy Service of the A Coruña University Hospital). This means 3190 doses for an average of 511 patients per year in Day Hospital; the necessary investment in technology integration to take action in this process seems therefore fully justified.

**Figure 1:**
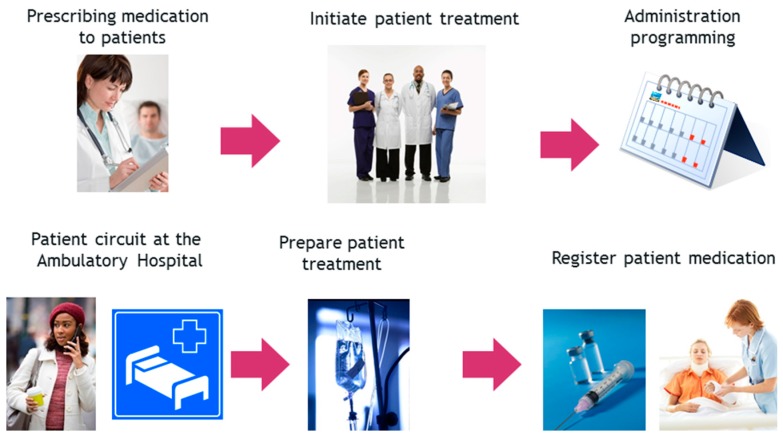
Plan of administration of medication in the Day Hospital.

The start-up of this work has not changed the essential functioning of prescription-validation-elaboration-administration process for patients in the Day Hospital. The plan is described in Figure 1.

- If the physician prescribes the medication to the patient for the first time, the authorization from the Hospital committee is needed. When authorized, patient treatment can proceed.
- Periodically, the physician schedules the future administrations to the patient.
- Before the patient arrives at the hospital, the pharmacist has validated and elaborated the preparation orders of the medication prescribed to the patient.
- In each scheduled administration, the patient goes to the doctor to check if he or she is in good health for treatment; if so, the doctor notifies the Pharmacy Service to start the preparation of the medication.
- Pharmacy staff develop and identify accurately the intravenous mixture to be administered to the patient.
- Finally, the nursing staff administer the medication to the patient: identify the patient and medication and check if everything has been carried out in compliance with all administration standards (dose, route, pattern, infusion rate, expiration date, etc.).
- Therefore, the development of systems responsible for obtaining traceability of patients and medication through the use of emerging and breakthrough technologies [8,9,10] such as RFID (the selection of RFID is justified in detail in following sections) and its integration into hospital staff daily practice, is essential.

## 3. RFID Technology (Radio Frequency Identification) and Other Identifying Technologies

We select the RFID technology [11,12,13] for several reasons:

- To provide an additional value to the WIFI network available in the hospital.
- To avoid the deployment of a new network of antennae (more expensive, implies interrupting the functioning of the hospital).

**Figure 2:**
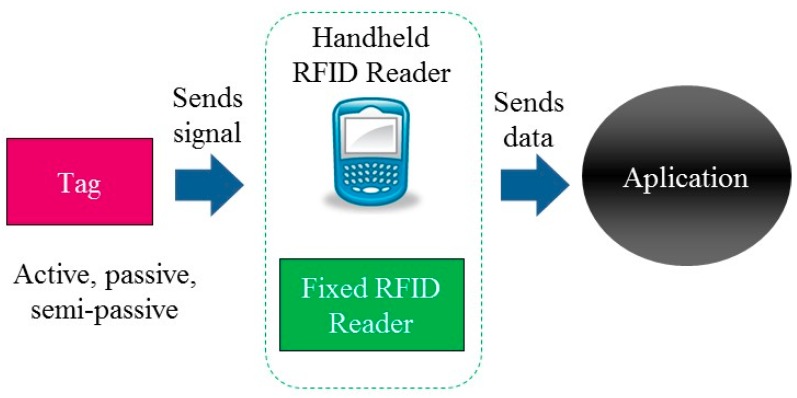
Operation of RFID technology.

In addition, RFID fulfills the requirements for the identification and location of the patients and medicines. RFID technology in recent years has been used in very different areas and has provided significant benefits. It is therefore interesting to study the viability of its integration into healthcare environments in order to improve patient safety and the quality of service received during the care process. The diverse characteristics of RFID technology components allow the establishment of a large number of different systems. However, the essential operation of RFID is fundamentally identical in all; a tag is attached to the object or person to be identified which then sends a radio frequency signal with the identifying information needed. A reader receives and transmits this data to a software application which then processes it, as seen in Figure 2.

Readers can be fixed (antennae, ARCHS, access points, etc.) or mobile (tablet, PDAs, phones, etc.). Tags can be active if they are equipped with their own energy source, or passive if they use the same energy source as the reader, and semi-active if their own energy is not used for transmitting but to power the internal circuitry of the microchip. There are other important differences between active and passive tags, such as cost, available sizes, and variations in coverage (higher in active and lower in passive) (See Table 2).

RFID component coverage is the maximum distance in which a communication can be established (to read or write data in its memory, change its settings, etc.). Apart from the operating frequency of the system, quality of coverage depends on factors such as the power provided by the reader antenna and available on the tag itself, the surrounding conditions, the orientation of the tag at the time of reading, etc. Therefore, the values previously assumed for each tag are merely indicative. There are four possible operating frequency of an RFID system, as shown in Table 3.

The superiority and success of RFID technology over other identifying technologies lies mainly in the ability of its tags to store high quantities of information on subjects and/or other items. In addition, this data can be saved according to standards set by EPCglobal [14]. The tags are not damaged by use thanks to the variety of resistant materials used in their construction such as epoxy, which can withstand extreme temperatures (as low as −21 °C) and hundreds can be read, written and updated simultaneously, without the need for direct contact with the reader. These properties make RFID the ideal technology for traceability systems in a healthcare environment (for cool/frozen or room temperature products, to identify patients, to use single doses of medication or infusion pumps, defibrillators, etc.). Tags are the most important components in RFID: their design is fully determined by the objects or subjects being tracked. There is a great variability of tags (shape and dimensions) and if the correct isolation is selected they can be read regardless of the material on which they are attached.

At present, several identifying technologies on the market could solve partially or completely the requirements outlined in this paper. Given the characteristics of this project, the selected technology must have the following functions:

- Unmistakably identify patients and medication.
- Estimate the location of a patient and/or an asset with good accuracy (1 to 4 m).
- Consist in physically comfortable devices during the patient’s stay at the hospital.
- Be suitable for a sterilization process.

This error range is acceptable and suitable considering the size of each of the areas to be controlled. It is essential to know if a patient is waiting or if he/she is being seen by a physician. Often different rooms are adjoining rooms and therefore location accuracy is key in determining that the system functions correctly and that the information provided is accurate. The Day Hospital is located on the ground floor of the CHUAC. We need this precision because on the same floor and adjacent to it are the patient waiting rooms, the medical area, and rooms dedicated to the administration of the medication to the patient. 

Having fulfilled the above requirements, the need to minimize disruptive construction work in the hospital and the reuse of existing infrastructure in the same (providing a new network value through already installed antennae) and therefore minimizing costs, are decisive in the selection of the technology that will address the development of this work. The following options were evaluated: ultrasound (Sonitor), infrared, ZigBee, and RFID. Table 4 shows the values of essential parameters for the proper functioning of the system to be developed in this work.

The conclusions that appear in the table are the result of our real experimentation with the indicated technologies [14]. We have tested different labels, frequencies, size of labels, distances of reading and/or writing for each of the technologies. This experimentation is key for the success in the design of the technical architecture of the system. After analyzing the above table, it can be said that RFID is the most suitable technology for the identification of drugs. This is due to the wide variety of shapes, sizes, and material of the tags, which can be adapted to almost any item/subject. Other technologies do not have adequately sized/shaped tags to identify unit doses of medication. One of the main disadvantages of RFID is the interference that can arise in the readings if the labels are attached to certain materials. In this work that obstacle has been overcome by attaching the tags in a “Flag Mode”, as detailed in the design section.

Regarding real time location, the accuracy of calculating the position of the patients has been essential due to the proximity of the different controlled areas and the use of a WIFI network in the hospital. The major disadvantage has been the high cost, along with the lack of automation in some of the necessary processes, which can be overcome with proper design. 

Recently RFID technology has been experimented with in healthcare environments. Many functions have been developed, such as the implementation of algorithms to improve communication between tags and RFID readers, RFID identification of patients to dispense electronically prescribed medication, mobile applications that facilitate monitoring of patients at their home addresses, location of assets and patients using active tags, passive tags, WIFI active tags, RFID automatic medication dispensers, etc. All these functions are intended to increase the patient care process safely, facilitating traceability of patients and prescribed/elaborated/dispensed/administered medication.

## 4. Development

### 4.1. Analysis

The first phase of project development is the analysis of the process of prescription-validation-elaboration-dispensing-administration of drugs in the Pharmacy Service and Day Hospital of the A Coruña University Hospital. The main objective was to study the performance of these services and discover the pathway medicines and patients follow from arriving at the hospital until they leave. This will enable detection of those critical points where the implementation of RFID technology is necessary to minimize the occurrence of adverse events and therefore increase safety in the patient care process.

**Figure 3:**
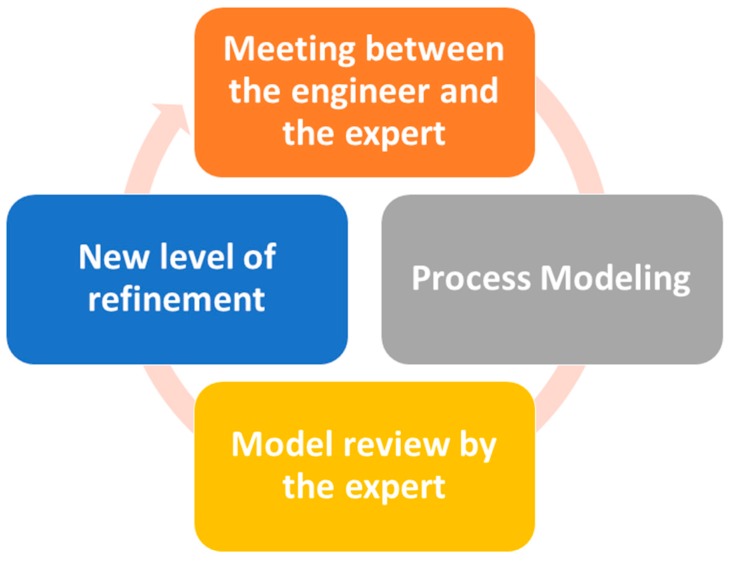
Analysis phase methodology.

The methodology used in this phase (as seen in Figure 3) is based on successive meetings with healthcare professionals as experts in each field. The aim of this phase is to acquire existing knowledge. This process is iterative and progressive, and with each iteration diagrams are obtained and presented for expert review. This way, medical staff can confirm that knowledge acquired is correct or can make suggestions for possible improvements. The software engineer builds more refined models in each iteration and facilitates the transmission of the specialist knowledge.

In order to present graphically all knowledge after the application of the methodology described, a tool called Architecture of Integrated Information System (AIRIS) was used. Its main target is to facilitate the development, implementation, and control of the business processes. Although it has different types of diagrams available, in this case the work protocol Chain of Processes guided by Events (CPE) is represented and allows to articulate any process in terms of events, functions, documents, jobs, control flows, and rules connection.

Events trigger functions and are a result of these. Functions indicate the activities carried out in the process at any time and the time spent on the same. Documents are objects representing documents that are used in the process, while position indicates which professional category is performing a function.

### 4.2. Design

This section is about describing and justifying the design of the RFID technical architecture necessary to achieve the objectives of this work, which were integrated in the Pharmacy Service and Day Hospital.

#### 4.2.1. RFID System for Traceability of Patients and RFID Transportation Cart

There are multiple solutions for tracking people and the transportation cart of intravenous mixtures prepared by RFID. This technology has several frequencies and each of them requires different components and can calculate the position with a certain precision. Table 5 shows a comparison of the major frequencies used for obtaining RFID traceability. The key parameters for the selection of RFID components in this work are:

- Calculating position with an accuracy of 1 to 4 m.
- Minimizing costs.
- Minimizing remodeling/construction works and thereby avoiding disruption of the normal operation of the hospital, and of course by making full use of the already available WIFI network in the hospital.

Analyzing the values of the parameters that can be seen in Table 2, active WIFI tags have been selected to locate patients and the medication cart, meeting the requirements defined. Currently there are two major competitors on the market for locating systems using active WIFI Real Time Location System (RTLS): Aeroscout and Ehakau. Aeroscout (Lincoln, NE, Nebraska) has been selected for this work for the following reasons:

- Higher localization accuracy.
- Active WIFI tags from both manufacturers have similar sizes, but Aeroscout is considered more comfortable for the patient in shape.
- Both systems support Cisco (the manufacturer of the WIFI network available in the hospital therefore minimizing the required infrastructure costs), but Ekahau requires an IP address for each tag and this could cause problems with the network management.

Table 6 shows a summary of the users, hardware components, and software of the RFID technical architecture.

**Figure 4:**
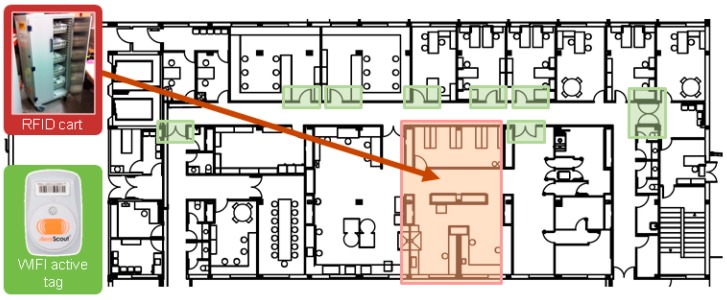
RFID System for Traceability of RFID Transportation Cart.

**Figure 5:**
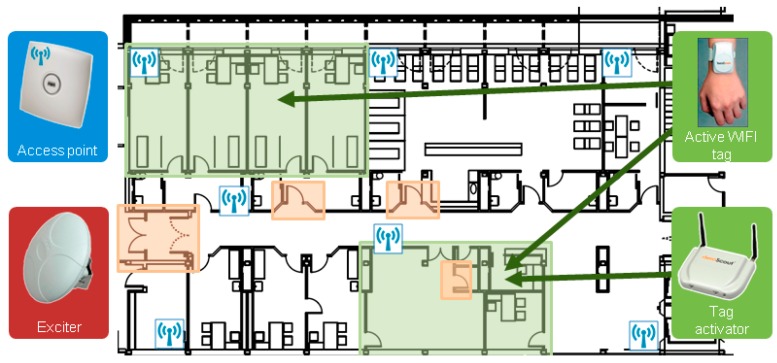
RFID System for Traceability of Patients.

These are the hardware components of the RFID system as seen in Figure 4 and Figure 5:

- Cisco Access Point, Aironet 1130 AG model: as a reference for calculating the position of the patient, at least three access points must simultaneously detect the patient to calculate his/her position. This manufacturer has been selected because the same WIFI network is already available in the hospital.
- EX2000B Exciter: to improve the calculation of the location accuracy. The main function of these devices is to communicate to the location engine which active WIFI tags are detected within their range. The engineer in the design phase is responsible for entering the physical location of exciters into the engine map location manually, so when an exciter indicates that it has detected a label, accuracy in the location calculation is high. Information obtained through the exciters can correct discrepancies in locations registered through the WIFI access points.
- Aeroscout Exciter Detector BWH-3000-SV: A device for applying Aeroscout Network Exciter Manager which monitors data concerning the coverage range and signal strength of exciters detected at all times.
- Tag Activator: A device that facilitates communication between Tag Management Suite application and AeroScout WIFI active tags (simultaneously detects one or more tags that are in the coverage range).
- WIFI active tag Aeroscout T2: to know the location of the patient and the cart.
- RFID cart: to transport and obtain the traceability of intravenous mixtures.

The RFID software components consist of the following:

- Mobile View: This application is proprietary to the Aeroscout manufacturer. It is the location engine interface and displays the traceability of any controlled asset. That is, the user can consult a graphic representing an individual patient for a period of time and his/her course during the stay in the hospital. It analyzes waiting time, processing, administration of drugs etc. and therefore facilitates the detection of bottlenecks and improves the operation of the service. It manages prohibited areas access alerts and abandonment by the patient of the hospital before drug administration (an action that should paralyze the elaboration of intravenous mixture in the Pharmacy). In addition, the user defines the buildings, floors and areas where the patients would be controlled [15].
- Cisco Mobility Service Engine MSE Locator 3350: calculates the position of patients using different algorithms and considering the access points that detect the tagged patient at all times; in this case the algorithm used is the RSSI which is intended for indoor location. This location engine has been selected because it is the one that requires the Aeroscout manufacturer for WiFi networks integrated by Cisco access points.
- Tag Management Suite: application property of Aeroscout manufacturer that enables, disables, and configures WIFI active tags parameters (frequency and broadcast channels, etc.).
- Aeroscout Network Exciter Manager NEM-1000: application property of Aeroscout manufacturer to manage and configure exciters in a Cisco LWAPP environment.

#### 4.2.2. RFID System for Medication Preparation in the Pharmacy Service

This system is responsible for the unmistakable identification of intravenous mixtures created in the Pharmacy Service and subsequently administered to patients in the Day Hospital. Table 7 shows the users, and the hardware and software components of the system.

**Figure 6:**
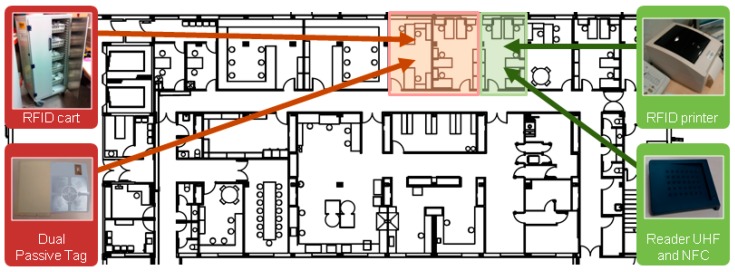
RFID System for Medication Preparation in the Pharmacy Service.

These are the hardware components of the RFID system as seen in Figure 6:

- Toshiba B-EV4D-GS14-QM-R Printer: Prints the tags that will identify the prepared intravenous mixtures.
- Dual passive tag with NFC (NXP NTAG203, ISO 14 443 A) + UHF (Monza 4, Operating Frequency 860–960 MHz, International Standards EPC Class 1 Gen 2, ISO 18000-6C): Passive tags made and designed at size 100 mm × 70 mm. The tag is attached to the intravenous mixture in the “Flag Mode” to prevent possible RFID interference with liquids, and contains in a single label two passive RFID tags in two different UHF and NFC frequencies. The UHF tag (this frequency has been chosen so it can be read within the RFID cart) will be detected in the RFID system for medication traceability instead; the NFC tag (this frequency was selected since the readings will be made between short distances) is designed to operate in the RFID system for medication administration to the patient. Both systems are described in detail below.
- RD 200-U1-G (UHF) y RD 200-M1-G (NFC) Reader: Once the medication is prepared and labeled, it is necessary to associate the tag that identifies the prepared mixture. To do this, two specific USB readers for each frequency automatically read the unique identification item (UID) of the NFC and UHF tags, by simply moving them closer to the readers. Both devices have the same design. It is important to note that these devices are difficult to acquire as they are the only models that do not need additional software development and they are able to write UIDs and EPC tag data in keyboard emulator mode. This has greatly expedited the implementation of this work.

There is an application that electronically records the process of prescription-validation-dispensation-administration of medication in the Pharmacy Service and Day Hospital that follows the Day Hospital RFID protocol.

#### 4.2.3. RFID System for Intravenous Mixtures Traceability

This system manages traceability of intravenous mixtures during transport, from elaboration at the Pharmacy Service until final delivery in the Day Hospital. When the nursing staff finish labelling, they enter the medication on the intravenous mixtures RFID cart for its administration to the patient in the Day Hospital. This cart is an innovating and differentiating factor, because with the antennae it contains inside it is able to provide a list of the medication it contains in real time and with a frequency of retransmission previously defined by the user. Its operating mode is based on the mass reading of UHF tags from medicines contained within. At the same time it provides information on the mixture (drug, dose, route, pattern, etc.) and patient (medical history number, name, service, etc.) to which each belongs.

The first time the cart detects the UHF tag that identifies a mixture, it automatically sets the date and time sent. Also this cart is characterized by having a WIFI active tag (the same as the patient) that facilitates the control of traceability in real time; therefore its position or route for a period of time can be checked at any time set in the Mobile View application, i.e., the RFID system allows the route used by the medication during transport from the Pharmacy Service to the Day Hospital to be monitored.

The complex construction of this cart is noteworthy, since it offers almost 100% reliability in readings and is able to read content only, without detection errors with external tags, regardless of how close they are. Without RFID technology this would be quite difficult to achieve.

When the cart has reached the Day Hospital it is essential to record the delivery of drugs to the nursing staff. To do this, the orderly must deposit mixtures above an RFID tray, also an innovating element, which displays data of the mixture and the patient for whom it has been prepared. At the same time the RFID system reads the UHF chip from that drug, including the time and date of delivery. It is important to note that together with the information provided by the RFID system described above, it can also provide real time location of any intravenous mixture since the content and location of the cart is known at all times. Table 8 shows the users, hardware components, and system software.

**Figure 7:**
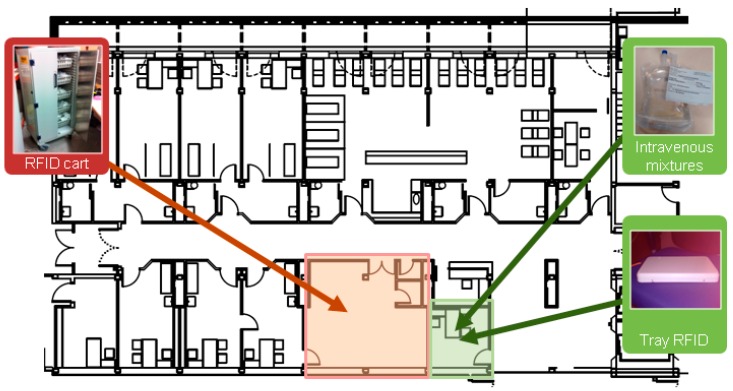
RFID System for Intravenous Mixtures Traceability.

The hardware components of RFID system as shown in Figure 7 are the following:

- Passive dual tag with NFC+UHF: tag for identifying intravenous mixtures.
- RFID cart: For transporting intravenous mixtures and obtaining traceability.
- Active WIFI tag Aeroscout T2: for the location of the cart.
- RFID tray: for controlling the delivery of medication in the Day Hospital.

The software components of the RFID system are:

- RFID Protocol for Day Hospital: application that electronically records the process of prescription-validation-dispensation-administration of medication in the Pharmacy Service and the Day Hospital.
- Cart RFID Real Time: application developed in the IT Service to display real time information on intravenous mixtures that are placed inside the cart (patient name, medication, dosage, etc.). The first time the cart reads a tag it sets the time of shipment of the intravenous mixture identified.
- Tray RFID Real Time: an application developed in the IT Service to display real time information on intravenous mixtures that are above the tray (patient name, medication, dosage, etc.). The first time the tray reads a tag, it sets the time for delivery of the identified intravenous mixture.

#### 4.2.4. RFID System for Medication Administration to the Patient

This system is responsible for the traceability and safety of medication administration to the patients in the Day Hospital. When the nursing staff administer the intravenous mixture to a patient, it is essential that they can check that the prescribed medication is administered in suitable conditions. To do this, the nursing staff (using a mobile device, an Android phone that has an integrated NFC reader) read the NFC chip from the intravenous mixture and subsequently they read the NHC from the patient. From that moment onwards the application issues alerts relating to the administration of the drug to the patient, such as: route, pattern, conditions of administration, intravenous mixture expiry date, and whether the drug has been prescribed or had been given to the patient previously, etc.

It is important to note that this process significantly increases patient safety by minimizing the occurrence of adverse events and thus improves the quality of the patient care process during the stay in the hospital. Table 9 shows the users and the hardware and software components of this system.

It is important to note that this process significantly increases patient safety by minimizing the occurrence of adverse events and thus improves the quality of the patient care process during the stay in the hospital. Table 9 shows the users and the hardware and software components of this system.

**Figure 8:**
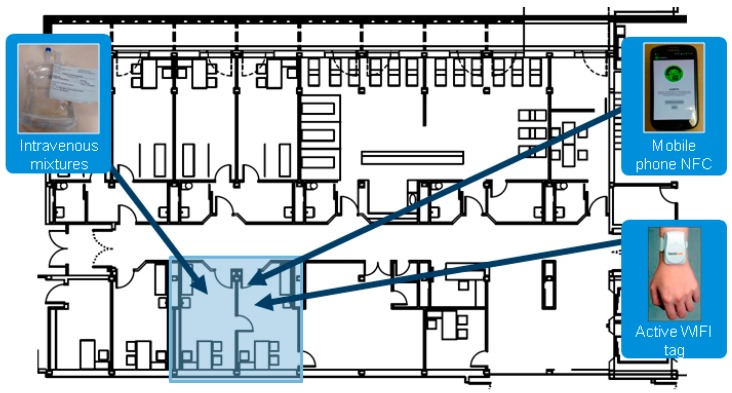
RFID System for Medication Administration to the Patient.

The hardware components of the RFID system, as shown in Figure 8, are:

- Dual passive tag with NFC + UHF: A tag to identify intravenous mixtures.
- Android mobile with NFC: in orer to check that the correct medication is administered to the patient. This device has been selected for its low cost within the range of mobile phones with integrated NFC readers. In addition, the screen size and interface usability minimize the training period with health professionals.
- Active WIFI tag Aeroscout T2: used to identify the patient.

The software components for this RFID system are the following:

- RFID Protocol for Day Hospital: an application that electronically records the process of prescription-validation-dispensation-administration of medication in the Pharmacy Service and Day Hospital.
- AndRFID: an application developed by the CHUAC IT Service for the nursing staff to check accurately and without error that the prescribed medication is administered to the patients, and in suitable conditions (route, dose, pattern, infusion rate, stability, expiration date, etc.).

### 4.3. Implementation

This section will describe how the implementation of RFID hardware and software (detailed in the above design section) was carried out. This phase is divided into different stages and each is characterized by the grouping together of tasks of different natures. Moreover, the stages are designed so their development is performed simultaneously and that the global time-scale of implementation can be expedited.

Software development has followed the standards defined by the Galician Health Service (Servizo Galego de Saúde, SERGAS) based on Metric V3.4, in order to ensure that the results have the highest level of quality possible and at the same time meet the standards set by SERGAS for corporative systems that will be put into daily clinical practice production.

It is important to emphasize that there is a software interface for the system in which all the events of traceability (generated by the different devices RFID and available as backend) can be registered, consulted, and analyzed by the users. The following section describes in detail each of these stages.

#### 4.3.1. Aeroscout RTLS

The main objective of this stage is to install and configure the Aeroscout manufacturer infrastructure for the traceability of patients, medications, or other items to be controlled by an RTLS system. More specifically, the RFID system for traceability of patients and the RFID transportation cart is described in the design phase. The first step consists in entering floor plans to control the engine location. In this case the floors are the following:

- Floor 1: Pharmacy Service.
- Level 0: Day Hospital, Cafeteria, hospital gateway and exit.

**Figure 9:**
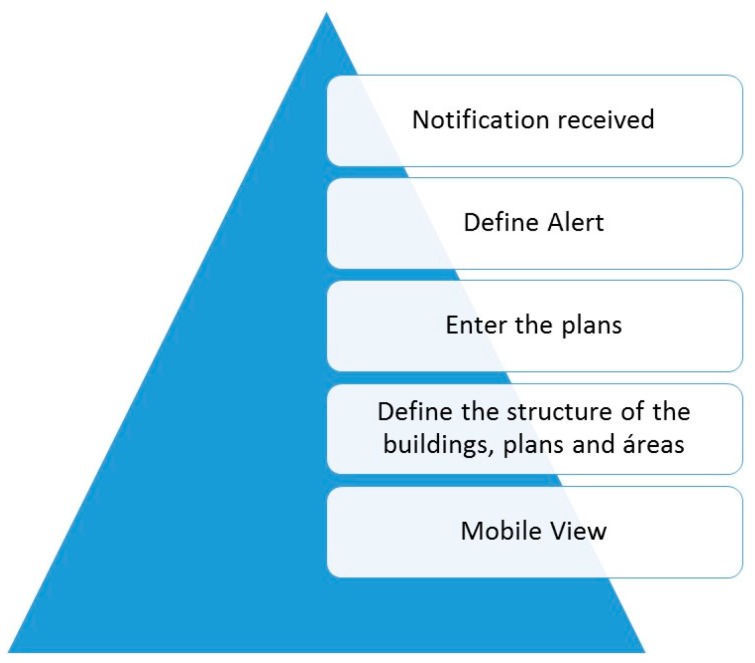
Mobile View tasks.

Later in the Mobile View, the following parameters must be set as seen in Figure 9.

- Define the structure of the buildings, floors, and controlled areas
- Enter the plans of each of the floors. Set additional possible alerts for: access zones, and access or exit alerts for certain group of tags.
- Decide what actions to take if alerts occur. These messages are sent as mobile texts messages and/or emails depending on user needs. In the following cases, alerts are always transmitted by emails to different health professionals: patient arriving/leaving the hospital, RFID cart reaching the Day Hospital or leaving the Pharmacy Service, etc.

Once the physical space is defined, we must carry out a coverage study in order to obtain the following data:

- The localization accuracy that can be obtained with the available WIFI infrastructure.
- How the WIFI network should be modified regarding the number of access points and their position in order to enable an RTLS accuracy of between 1 and 4 m.

The localization accuracy that can be obtained with the WIFI infrastructure available. How the WIFI network should be modified regarding the number of access points and their position to enable an RTLS accuracy of between 1 and 4 m.

The parameters that have determined this report are the following:

- Physical location of each of the access points.
- Physical location of the antennae.
- Types of antennae.
- Frequencies and channels used for each access point.
- Transmission power of each point.
- Signal range for each access point (signal strength in the environment) SNR (Signal-to-Noise Ratio).
- Possible sources of interference (elevators, wall material, types of rooms: open space, semi open space, etc.).

Material needed is described below:

- WIFI access point (Airlap 1131ag-e-k9 from Cisco).
- Ekahau Site Survey 2.1 tool for taking sizes.
- Laptop that includes a WIFI card.
- Plans for areas to be controlled.

The procedure has been integrated from the tasks described below:

- Theoretical study of coverage or simulated by software
- Study of actual coverage
- Analysis of the study of actual coverage and design of WIFI network.
- Measurements taken in the study of coverage of the new WIFI network.
- Conclusions and writing the document

**Figure 10:**
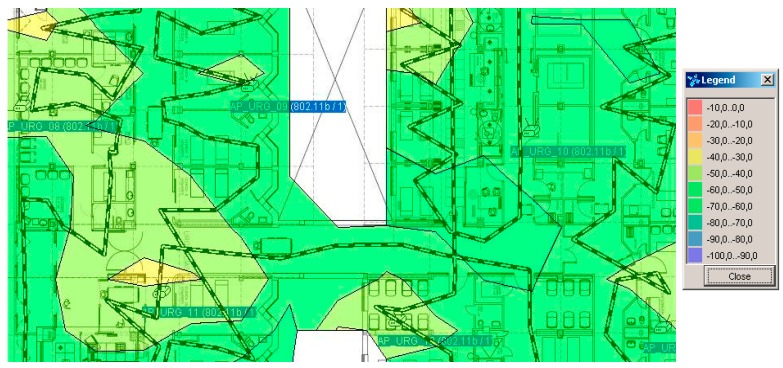
Power of the WIFI in each of the zones.

Figure 10 shows the data obtained in the study of system coverage prior to the installation of the system. 

The study of coverage is composed by the following phases:

1. Analysis of the study of coverage theoretical or simulated for software
2. Analysis of the study of coverage a real study.

The study of real coverage detected that the power of the WiFi signal was not as strong as the software was indicating. This problem was solved by approaching the points of access.

The power of the tags must be −75 dB as the Aeroscout manufacturer recommends. 

Figure 10 shows the power of the WiFi and the legend indicates the power of WIFI in each of the zones. From the study of the coverage system it is concluded that nine WIFI access points are needed, as shown in Table 10. 

In general, a RTLS system is not 100% accurate. In order to improve accuracy in localization, the manufacturer Aeroscout facilitates the possibility of installing exciters. The operation of these devices is simple: when an exciter detects a tag within their range of coverage it relays it to the location engine (associated with the location of the tag to the exciter) and thereby greatly improves the accuracy of the system in calculating location based solely on network WIFI access points.

To enable this, the engineer should manually indicate in the available plans of the location engine the placement and location of the exciters. In order to configure the exciters, an initial survey must be carried out by the Aeroscout Exciter Detector BWH-3000-SV hardware and AeroScout Network Exciter Manager NEM-1000 software. This process aims to determine which is the most appropriate range for each of the covered exciters (for this model, between 0.5 and 12 m in diameter is possible). Table 11 shows the number and the location of the exciters installed to significantly improve the accuracy of RTLS system. 

With the infrastructure detailed above, the RTLS system can calculate a position with an accuracy of 1 to 4 m. When the device detects a tag in an area controlled by exciters, it locates the tag in that area unmistakably until another exciter relocates it again in a new area. This corrects any errors in the network access points and allows the RTLS system to have, in most cases, an almost exact precision.

#### 4.3.2. RFID Protocol in Day Hospital

This work is focused in the prescription-validation-elaboration-dispensing-administration process of medication in the Day Hospital. A hub of information concerning traceability of the medication is required from the point of prescription of medication by the physician until the nursing staff administer the medication to the patient.

To achieve this we have used an application already in use in daily clinical practice within several hospital services [16] called SiMON (Intelligent Monitoring System), which is implemented in Java utilising the Informix database. This software allows users with an administrator profile to define a protocol primarily composed of a main registry and monitoring actions. Both are characterized by parameters (only in the case of the main registry the parameter structure will not change over the lifetime of the protocol) defined by the administrator that meet the needs of future users. One of the main advantages of SiMON is that it is integrated with the corporate applications SERGAS such as IANUS (responsible for EHR) or Laboratory (which manages different tests on patients).

This means that the parameters of the main registry or monitoring actions can be data-managed on a daily basis in the clinical practice of the hospital and a duplication is not required, which improves the quality, efficiency, and safety of the patient care process.

In this case the RFID protocol in the Day Hospital has been created with the following characteristics:

- Each patient has a unique principle record with invariable parameters: name and surname, NHR (Number of History Record), date of birth and age.
- Each main registry has monitoring actions and each one corresponds with a schedule for the administration of the medication made by the physician.
- Each monitoring action consists of six tabs with group information on each of the following threads: Date of administration, Prescription, Validation, Treatment Confirmation, Medication Elaboration, and Confirmation of the Administration.

Below are detailed the various tasks that can be carried out by different users under the professional profile in the RFID protocol of the Day Hospital:

Doctor:

- Prescribes medication electronically.
- Electronically schedules future administrations of the treatment.
- Records if the patient’s health is in proper condition for the administration of the drug.

Pharmacy staff:

- Validate the prescribed medication.
- Prepare elaboration orders for the nursing staff.
- Generate reports about monthly/yearly, etc. consumption for each medication, service, or both.
- Check the pending planning for a certain period of time.

Nursing staff in the Pharmacy Service:

- Have a consultant guide for the elaboration of the intravenous mixture.
- Visually print RFID tags.
- Identify unmistakably the intravenous mixture using RFID tag (records lot, date of expiry of its components, and the prepared medication).
- Bind the unique identifier (UID) from the UHF tag and the NFC tag that make the dual RFID tag which identifies intravenous mixtures.
- Record time of elaboration and shipping from the Pharmacy Service to the Day Hospital.

#### 4.3.3. Tray RFID Real Time

This application is developed and owned by the manufacturer responsible for the design of the RFID tray. It identifies the UID of the UHF tags of dual RFID labels deposited above it by sending an XML file with that information to a specific URL. It has been integrated with the RFID Day Hospital protocol by web services so that the first time the RFID tray reads an intravenous mixture its delivery time has to be set in the Elaboration of Medication corresponding tracking tab, taking into consideration the administration date that was already scheduled. 

#### 4.3.4. Cart RFID Real Time

This application is developed and owned by the manufacturer responsible for the design of the RFID transportation cart. It identifies the UID of the UHF tags of the dual RFID labels that are inside the cart, by sending an XML file with that information to a specific URL. It has been integrated with the RFID Day Hospital protocol by web services in such a manner that the first time RFID cart reads an intravenous mixture, its time of departure from the Pharmacy Service is set in the Elaboration of Medication corresponding tracking tab, taking into consideration the administration date that was already scheduled. 

#### 4.3.5. Configuration of USB Mode RFID Readers

USB mode readers must be configured so that there is consistency in reading dual information from RFID labels in all devices available in different RFID systems. Two models were found (UHF and NFC) that are operated by a keyboard emulator so that further development is not necessary to implement their functionality. Both should read 16 characters from UID tags and in hexadecimal format. The configuration of both readers cannot easily be confused and is performed by a desktop application created by the manufacturer of the devices.

#### 4.3.6. AndRFID

The AndRFID application has been developed in the CHUAC IT Service. Its main objective is that nursing staff can accurately check that the correct medication prescribed to the patient will be administered in suitable conditions (route, pattern, infusion rate, lot number and expiration date of the medication, etc.). It has been implemented in Java with Android and the interface design is easy and intuitive so as to minimize the training time of future users and to ensure service efficiency.

#### 4.3.7. Project Costs

The costs of the infrastructure WIFI have been minimal because the network (net) WIFI was available in the hospitable center. In the following table the most significant costs are described. It is important to emphasize that one part of this work was financed through project PI10/02442 of the Institute of Health Carlos III (around 40%) and that another part was financed by the hospital.

### 4.4. Evaluation of the System

The preparation phase lasted approximately four months and was necessary to engage the procedures of the Pharmacy Service, the Day Hospital, Rheumatology, and IT support. The meetings were organized every two weeks and in attendance were the heads of service and at least one representative of the professional profiles involved: a pharmacist specializing in hospital pharmacy, nursing staff from the Pharmacy, nursing staff from the Day Hospital, practitioners from Rheumatology, and software engineers from the R&D Systems Branch Information Systems from CHUAC.

**Figure 11:**
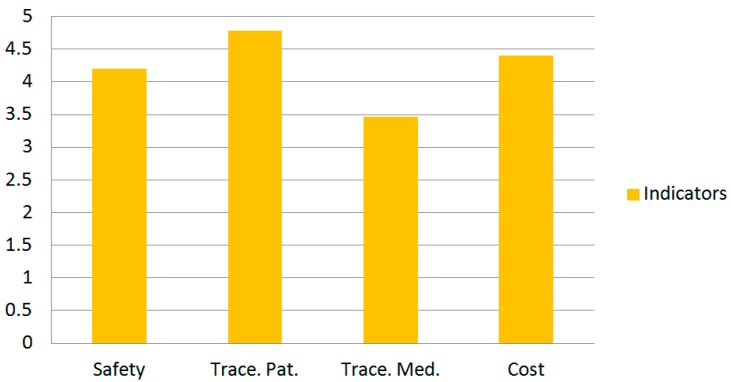
Global Values of the analysed Indicators.

On 4 December 2014 the first active WIFI RFID tag was delivered to a patient. On 19 January 2016 a total of 2515 intravenous prepared mixtures and 285 patients are managed through this project. To evaluate the system, seven questionnaires were designed with one for each professional profile (patient, doctor, pharmacist specialising in hospital pharmacy, nursing staff in the day hospital, computer personnel, subdirector of hospital systems) involved in the development and practical use of the system under evaluation. Only simple questions were presented, only related to tasks with which the professionals are directly involved so that, with their experience, they are likely to respond more accurately. The thematic content of the questionnaires evaluates some indicators of the systems: Safety, Traceability of patients, Traceability of medication, Cost. We analyzed in detail the values of the answers of 19 questionnaires (0: minimum level of satisfaction and 5: maximum level of satisfaction). The results of the first phase of evaluation indicates that the values of all the indicators are between 3.5 and 5 (see Figure 11).

At present, we are working on the evaluation of additional indicators such as Usability, Efficiency, and Suitability for Routine clinical practice. During the pilot test no important incidents happened. The principal incidents consisted in that the nursing staff sent the medication prepared for the Day Hospital before the doctor had confirmed the perfect state of health of the patient. It is necessary to emphasize that during the first days an average of six patients forgot the active WIFI tag RFID at their home.

One of the first agreements was to acquire the material described in Table 12, taking into account the volume of patients expected to meet the criteria for inclusion. Later, the successive meetings mainly dealt with the following points:

- Determine the new patient course: Arrivals and Departures from the hospital, waiting area, and Cafeteria.
- Elaboration of informed consent for patients.
- Circuit of delivering, activating, configuring, and collecting active tags.
- Determine training sessions for each healthcare professional (two hours a day during a week at the most).

Finally, there were a number of periodic tasks relating to active WIFI tags maintenance (for the RFID cart and patients) that were assigned to IT support:

- Management of low battery alert in active WIFI tags (collection and change).
- Configuration of active WIFI tags in first time use (battery test, number of channels, frequency of transmission).

**Figure 12:**
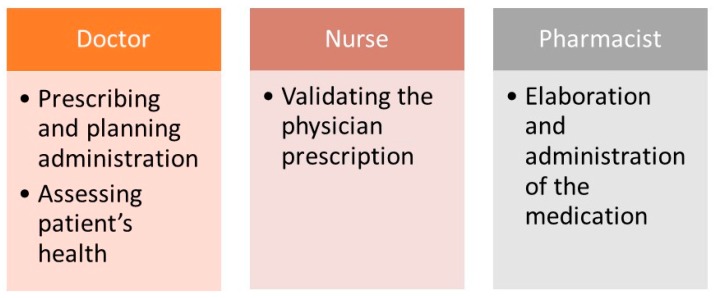
Cases of use by health professionals.

The start-up of this effort has not changed the essential functioning of prescription-validation-elaboration-administration process of medication to patients in the Day Hospital. The following describes the steps and summarizes the main cases of use by health professionals, as seen in Figure 12.

If the physician prescribes a medication for the first time to a patient following the RFID protocol for Day Hospital, authorization is needed from the hospital commission. If authorized, the treatment starts and the physician explains how the system works through informed consent, and if the patient accepts, the physician gives the active WIFI tag from Aeroscout to the patient. 

**Figure 13:**
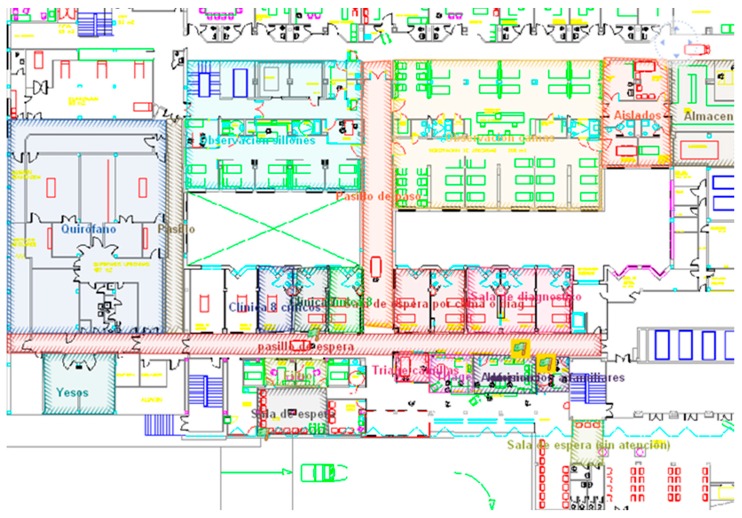
Location in real time of the patient.

The physician binds the NHR to the tag MAC which localizes it in Mobile View, to later track the patient in the system (see Figure 13). This means that in each of the administrations scheduled by the doctor, there is real time knowledge on the arrival and departure of the patient to and from the hospital as well as on his or her location in real time on each of the controlled areas, i.e., administration room in the Day Hospital, cafeteria, waiting room, etc.

Periodically, the physician programs future administrations of medication in the Date of Administration tab in the RFID protocol for the Day Hospital. Before the patient arrives at the hospital, the pharmacist has validated and elaborated the preparation orders of the medication prescribed to the patient. In each administration scheduled, the patient goes to the doctor to check if he or she is in good health for treatment; if so, the doctor notifies the Pharmacy Service through the Treatment Confirmation tab, following the RFID protocol for Day Hospital.

**Figure 14:**
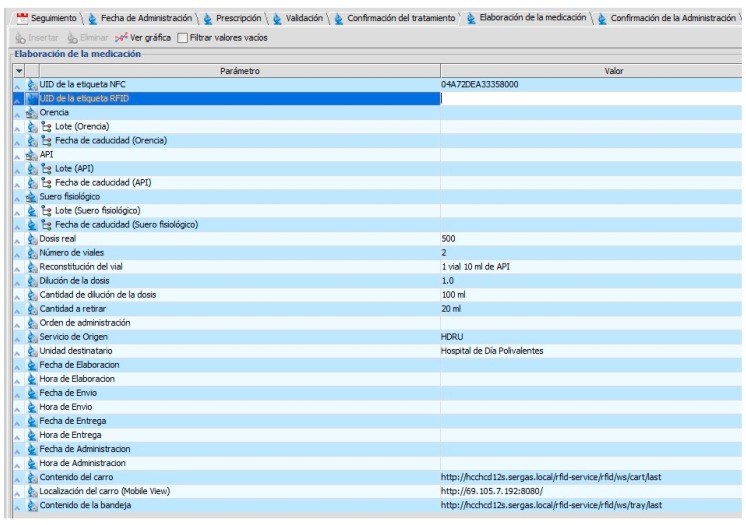
Traceability of the elaboration of the intravenous mixture.

Pharmacy staff develop and unmistakably identify the intravenous mixture to be administered to the patient through the Medication Elaboration tab following RFID protocol for the Day Hospital (see Figure 14) and the RFID system for preparing the medication in the Pharmacy Service. Later, the medication is placed in the RFID cart; by using the RFID system of medication traceability it can be tracked during its transport from the Pharmacy Service to the Day Hospital. 

Finally, when the cart arrives to the Day Hospital, the orderly places the medication in the RFID tray to control its delivery and the nursing staff administer the medication through the RFID system for administrating medication to the patient. This identifies the patient by reading his or her NHR and reads the tag from the medication and the mobile application by different alerts. It also confirms that the prescribed medication is going to be administered to the correct patient and in suitable conditions (dose, route, pattern, infusion rate, expiration date, etc.).

The number of patients included increases monthly as the objective is to cover the total number of patients who have prescription medications that are considered high risk and cost and are administered in the CHUAC Day Hospital. The most important incidents detected so far have been: problems in reading the bar code of the MAC tag (misdirection in the reader), and error in the location of the attachment of the RFID tags on intravenous mixtures prepared by medical personnel. All of these have been progressively minimized as the learning curve of the medical staff has improved. It is important to note that the system is prepared to be easily extended to any other service/treatment prescribed in CHUAC.

## 5. Conclusions

In conclusion, RFID technology has been successfully integrated in the process of prescription-validation-elaboration-dispensation-administration of drugs in the Pharmacy and Hospital CHUAC, significantly increasing patient safety by minimizing the possible occurrence of adverse events. As seen above, RFID systems that work simultaneously in clinical practice have been developed and utilized successfully. These have varying and very different technical features: such as obtaining medication and patient tracking by designing a RFID cart and a RFID tray that unmistakably identifies patients and medication; but these features have been integrated with each other to a high standard of success. It should be underlined that this system and study has overcome one of the main difficulties inherent in this work: the fact that the design of its technical architecture features a combination of several operating frequencies (UHF control drugs in the car and RFID tray, NFC for administration by mobile phone) and several different energy sources for the tags (WIFI active, passive), all of which must function collectively.

Importantly, in this work, we have solved some problems that are intrinsic to the nature of the technology such as readings on liquids in glass or plastic containers. It is possible to read 100% of the drugs labelled due to the strategic location in “Flag Mode” of passive tags and RFID that prevents interference with certain materials. To ensure the security of data stored on tags, the UID (unique identifier factory) has been used and is associated in the database to the prescription and clinical data of each patient. Another important element is that the location precision components manufactured by Aeroscout is accurate between 1 and 4 m in all controlled areas of the Pharmacy Service and Day Hospital.

This study has concluded that the integration of RFID technology in the healthcare environment is economically viable, in particular for the selected scenario, considering the high risk characteristics of the medication and diseases for which these treatments are prescribed. It can be seen that RFID can significantly increase the quality of care received by patients during their care process in this scenario. Regarding immediate future work, the evaluation of the system by health professionals involved in their use in clinical practice is proposed, so as to enable improvements in the potential weak points detected.

## Figures and Tables

**Table 1:** Key parameters in intravenous mixture.

Key Parameters
Patient’s data
Composition
Elaboration time
Pharmacist in charge
Expedition time
Health professional who prepared the medication
Stability
Special conditions of use
Previous medication
Infusion Rate
Lot and expiry of the components
Lot and expiry of the final product
Medication prepared

**Table 2:** Comparison of RFID readers and tags.

Fixed Reader	Mobile Reader	Active Tags	Passive Tags
Antennae	Tablet	Battery	No Battery
Arches	PDA	Higher cost	Lower cost and coverage
Access points	Mobile phone	Larger size	Longer lifetime

**Table 3:** RFID technology frequency of operation.

Frequency of Operation	Band
Low Frequency	Below 135 kHz
High frequency	13.56 MHz
Ultra High frequency	433 MHz, 860 MHz and 928 MHz
Microwaves	2.45 GHz and 5.8 GHz

**Table 4:** Comparison of different identifying technologies.

Feature	Ultrasounds (Sonitor)	Infrared (NPS ActiveTrack)	RFID (UHF, HF, NFC, Ekahau, Aeroscout)	Zigbee (Awarepoint)
**Unmistakably identification of the patient**	Yes	Yes	Yes	Yes
**Unmistakably identification of the medication**	No	No	Yes	No
**Accurate location between 1 and 4 m**	No	No	Yes	No
**Location at area level**	Yes	Yes	Yes	Yes
**Autoclave sterilization process**	No (Disposable case tags)	No	No	Yes
**Readings interference, attached to certain materials (metals, liquids in plastics and glass)**	No	No	Yes	No
**Comfortable device for the patient**	Yes	Yes	Yes	Yes
**Possible reuse of hospital WIFI infrastructure**	No	No	Yes	No

**Table 5:** Different methods for obtaining traceability with RFID.

Feature	Active Tags	WIFI Active Tags	UHF Passive Tags
**Accurate location between 1 and 4 m**	No	Yes	No
**Location at area level**	Yes	Yes	Yes
**Possible reuse of hospital WIFI infrastructure**	No	Yes	No

**Table 6:** Technical architecture of the RFID system in tracking patients and the medication cart.

Users	Hardware	Owned Software
Medical staff	Cisco access points, Aironet 1130 AG model	Mobile View
Pharmaceutical Staff Hospital Pharmacy Specialist	Exciters EX-2000B	Location engine Cisco MSE Locator 3350
Nursing staff from the Day Hospital	AeroscoutExciter Detector BWH-3000-SV	Tag Management Suite
	TagActivator	Aeroscout Network Exciter Manager NEM-1000
	WIFI active tag Aeroscout T2	

**Table 7:** Technical architecture of the RFID system in the preparation of medication in the Pharmacy Service.

Users	Hardware	Software Developed in the IT Service
Nursing staff from the Pharmacy Service	Toshiba B-EV4D-GS14-QM-R Printer	RFID protocol for Day Hospital
	Dual Passive Tag	
	Reader RD 200-U1-G (UHF)	
	Reader RD 200-M1-G (NFC)	

**Table 8:** Technical architecture of the RFID system for intravenous mixtures traceability.

Users	Hardware Components	Software Components Developed in the IT Service
Medical staff	Dual passive tag	RFID protocol for Day Hospital
Pharmaceutical Staff Hospital Pharmacy Specialist	RFID transportation cart	Cart RFID Real Time
Nursing staff from the Day Hospital	Aeroscout T2 WIFI active tag	Tray RFID Real Time
	RFID tag	

**Table 9:** Technical architecture of the RFID system in the administration of medication to the patient.

Users	Hardware Components	Software Components Developed in the IT Service
Nursing staff from the Day Hospital	Dual passive tag with NFC + UHF	AndRFID
	Aeroscout T2 WIFI active tag	RFID protocol for Day Hospital
	Mobile phone with integrated NFC	

**Table 10:** Installed WIFI access points.

Number of Access Points	Location	Areas Controlled
7	Ground Floor	Hospital gateway/exit door Day Hospital gateway/exit door Cafeteria Patients waiting room Medication administration room Medical consultation room
2	Floor 1	Pharmacy Service

**Table 11:** Installed exciters.

Number of Exciters	Location	Areas Controlled
4	Ground Floor	Hospital gateway/exit door Day Hospital gateway/exit door Cafeteria Medication administration room
1	Floor 1	Pharmacy Service gateway/exit door

**Table 12:** Cost of the Materials for the System and System Test.

Material	Cost/Unit
Android mobile phones with NFC (3 units)	150 euros
USB mode UHF reader (2 units)	200 euros
USB mode NFC reader (2 units)	200 euros
Printer (2 units)	3,000 euros
RFID Tray (1 unit)	3,000 euros
Software Aeroscout of RTLS Systems (1 unit)	18,327.11 euros
Laptop (1 unit)	700 euros
RFID Transportation Cart (1 unit)	7,913.47 euros
RFID passive tags (500 units)	1 euro
RFID Aeroscout WIFI active tags (200 units)	70 euros
Lanyards for the patient to carry the tags (200 units)	0.3 euros
Bags with eurotaladro for each WIFI active tag (500 units)	0.01 euros
